# The Use of Patient-Oriented Mobile Phone Apps in Oral Health: Scoping Review

**DOI:** 10.2196/46143

**Published:** 2023-09-06

**Authors:** Elina Väyrynen, Sanna Hakola, Anniina Keski-Salmi, Hannaleena Jämsä, Raija Vainionpää, Saujanya Karki

**Affiliations:** 1 Research Unit of Population Health Faculty of Medicine University of Oulu Oulu Finland

**Keywords:** oral health, dentistry, mobile apps, mobile health, mHealth, mobile phone

## Abstract

**Background:**

Oral health is a significant part of general health. Poor oral health can influence an individual’s appearance, self-esteem, eating, and speaking. The use of mobile phone apps has been growing in the field of medicine, including dentistry. However, to date, there is no evidence related to the availability of mobile apps focusing on various branches of dentistry.

**Objective:**

The aim of this study was to review the scientific literature on the use of patient-oriented mobile phone apps in oral health and summarize the key findings.

**Methods:**

A scoping review of published scientific literature on the use of patient-oriented mobile phone apps in oral health was conducted in accordance with the Joanna Briggs Institute. A search was performed in PubMed and Scopus for studies published between January 2000 and June 2021 that were written in English. All study types except for those reporting developmental protocols were included in this review. In total, 2 reviewers independently screened the studies using the eligibility criteria. The study protocol was registered in the Open Science Framework registries in June 2021.

**Results:**

The initial search yielded a total of 977 studies, 45 (4.6%) of which met the inclusion criteria. All the studies (45/45, 100%) were published after 2009. Most studies (31/45, 69%) concerned oral health promotion using mobile phone apps, followed by behavior management (5/45, 11%). More than half (23/45, 51%) of the included studies were conducted in Asian countries. Overall, 31% (14/45) of the studies focused on adolescents. A total of 51% (23/45) of the studies were randomized controlled trials (RCTs). Approximately 39% (9/23) of the included RCT studies reported a substantial reduction in dental plaque, and 26% (6/23) of the studies reported significant improvement in gingival health. Regarding dental anxiety management, 13% (3/23) of the RCT studies reported a significant decrease in mean heart rate and lower Facial Image Scale scores.

**Conclusions:**

According to the literature, the use of mobile apps in oral health is increasing among patients, mainly children and adolescents. Many studies that have used mobile apps have focused on promoting oral health. However, other areas such as diagnostic and remote consultations (teledentistry) have until recently been neglected despite their great potential.

## Introduction

### Background

Oral health is a significant part of general health. Poor oral health can influence an individual’s appearance, self-esteem, eating, and speaking [1]. Poor oral health is also one of the leading causes of loss of work productivity, increased school absenteeism, and reduced academic performance [2,3].

The risk factors for the most common oral diseases, such as dental caries and periodontal diseases, include physical, biological, environmental, behavioral, and lifestyle-related factors [4,5]. Most of the behavioral and lifestyle-related factors responsible for oral diseases are also common to various systemic diseases [6]. For instance, frequent consumption of foods and drinks containing free sugar can lead to dental caries as well as obesity [7]. Similarly, tobacco use is a risk factor for periodontal diseases, cardiovascular disease, respiratory diseases, and cancer [6,8]. In addition, poor oral health has an adverse effect on individuals’ general health. For example, periodontitis is considered one of the risk factors in the pathogenesis of diabetes mellitus, cardiovascular disease, kidney disease, and recurrent pneumonia [7,9]. Despite being preventable, >3.5 billion people have been affected by oral diseases worldwide, predominantly dental caries and periodontal diseases [10]. Although the concept of managing oral diseases has shifted toward prevention at the individual level, actions are being taken to move toward a more patient-centered management of oral diseases, focusing on promoting and maintaining good oral health in partnership with the patient [11].

Health promotion has been a key component in disease prevention. Behavior change approaches, such as communicating disease risk information and the self-monitoring of one’s own health as part of health promotion, have been successful in modifying the health behavior of individuals. Behavior change approaches refer to the specific strategies used in interventions to promote behavior change [12,13]. Behavioral interventions involve people’s decision-making process about their health.

Recently, the use of mobile technologies in the medical field (also known as mobile health [mHealth]) has been growing. With their many features and high use by consumers, mobile phones are one of the devices suitable for accessing health information, improving the quality and coverage of health care, and promoting health [14,15]. In addition, the use of mobile phone apps has been shown to have positive outcomes in general health. For instance, mobile apps are used for remote consultation [16], disease diagnosis [17], reminders for patients [18], and behavior modifications [19].

As of 2017, there were >325,000 mobile apps available on the Google Play Store and Apple App Store platforms that mainly focused on health and well-being [20]. Similarly, in dentistry, a total of 1075 oral hygiene apps were available as of 2018 [21]. Evidence on the availability of mobile apps focusing on various branches of dentistry is still unclear. In addition, with mHealth being popular, it is important for health professionals to know the availability of different kinds of mobile apps and be assured that the mobile apps are disseminating fact-based information [22]. Therefore, the aim of this study was to examine the main findings in the literature on the use of patient-oriented mobile apps in oral health.

### Review Questions

The primary question is as follows: What are the main findings in the literature related to the use of patient-oriented mobile apps in oral health? The secondary questions are as follows: What types of evidence are available in the literature related to the primary question? and How has this research been conducted?

## Methods

### Information Sources and Search Strategies

This scoping review was conducted in accordance with the Joanna Briggs Institute methodology for scoping reviews [23]. A preliminary search of MEDLINE, the Cochrane Database of Systematic Reviews, and *JBI Evidence Synthesis* was conducted on January 22, 2021, and no current or ongoing systematic or scoping reviews on the topic were identified. The text words contained in the titles and abstracts of relevant articles and the index terms used to describe the articles were used to develop a full search strategy for PubMed and Scopus. The search strategy, including all the identified keywords and index terms, was adapted for each database included. The reference lists of all included sources of evidence were screened for additional studies. The search terms used were “Mobile Applications” (Medical Subject Headings [MeSH]) OR “Mobile Application*” OR “Mobile App*” (text word) OR “Mobile health” (text word) OR “mHealth” (text word) OR “Health app*” (text word) OR “smartphone app*” (text word) AND “Dentistry” (MeSH) OR “dental*” (text word) OR “Oral Health” (MeSH) OR “Oral Hygiene” (text word) OR “Oral Medicine” (text word).

The study protocol was registered in the Open Science Framework registries in June 2021 [24].

### Eligibility Criteria

#### Overview

This scoping review considered all study types (experimental and quasi-experimental, cohort, case-control, cross-sectional, case series, case reports, descriptive cross-sectional studies, systematic reviews, and both qualitative and quantitative studies) that were published between January 2000 and June 2021 in English. Studies focusing on the development of mobile apps related to oral health were excluded. The participants, concept, and context guides for this scoping review are as follows.

#### Participants

This study included all age groups.

#### Concept

Patient-oriented mobile apps that are used in oral health were included.

#### Context

All areas, cultures, and sexes were included.

### Study and Source of Evidence Selection

Following the search, all identified citations were collated and uploaded into the Covidence data screening and extraction tool, and duplicates were removed. To ensure the calibration, a pilot test was arranged. A total of 25 samples of titles and abstracts were selected for the pilot test by one of the team members (SK), 2 reviewers (AK-S and SH) conducted the pilot test using the eligibility criteria, and the agreement was 84% [23]. Following the pilot test, titles and abstracts were screened by 2 independent reviewers (AK-S and SH) for assessment against the inclusion criteria. Full texts of potentially relevant sources were retrieved, and their citation details were imported into the Mendeley Reference Manager (Mendeley Ltd). The full texts of selected citations were assessed in detail against the inclusion criteria by 2 independent reviewers. The reason for excluding sources was that the full text did not meet the inclusion criteria of this scoping review. Any disagreements between the reviewers at each stage of the selection process were resolved with an additional reviewer (SK). The results of the search and the study inclusion process were reported in full in the final scoping review and presented in a PRISMA-ScR (Preferred Reporting Items for Systematic Reviews and Meta-Analyses extension for Scoping Reviews) flow diagram [25].

### Data Extraction, Data Analyses, and Risk-of-Bias Assessment

The extracted data included specific details about the participants, concept, context, study methods, and key findings relevant to the review questions. On the basis of the extracted data, the proportions of year of publication, subgroups based on focus areas, and study types were calculated and presented in a table.

No risk-of-bias assessment was performed in accordance with the Joanna Briggs Institute methodology for scoping reviews [23].

## Results

### Selection of Studies

**Figure 1:**
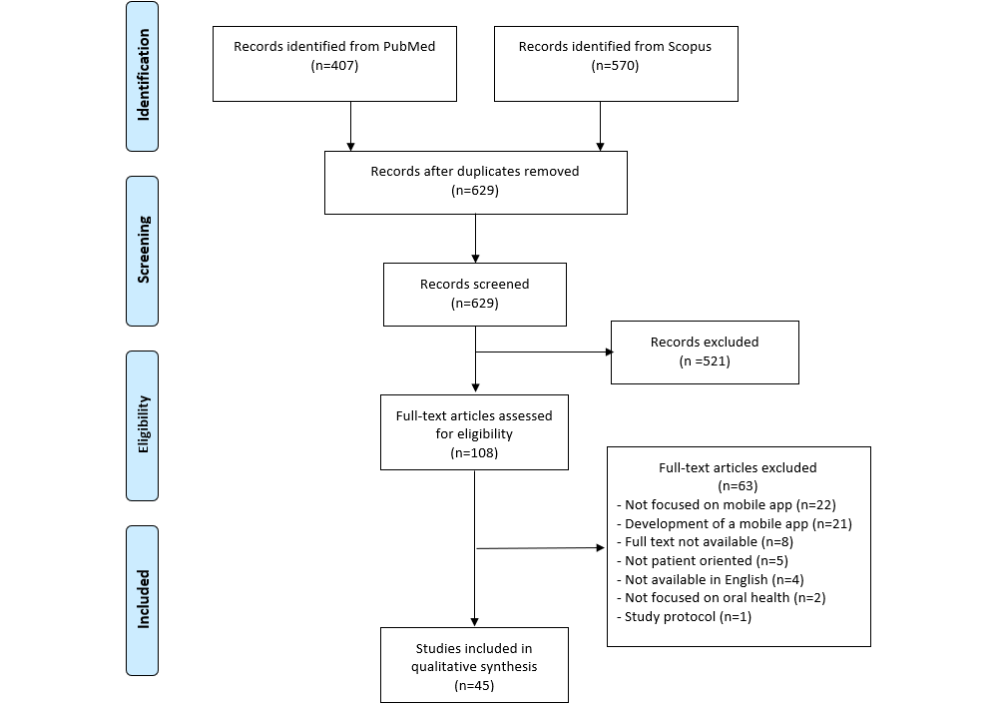
PRISMA (Preferred Reporting Items for Systematic Reviews and Meta-Analyses) flowchart of study selection progress.

A total of 977 studies were found in the initial search, as shown in Figure 1. After removing duplicates, 64.4% (629/977) of the studies were screened. Studies that did not meet the inclusion criteria were excluded. The most common reasons for exclusion were that the study was not related to oral health, it did not include a mobile app, the full text was not available, or the study was not written in English. A total of 45 studies were included in the scoping review.

Data were extracted from the included studies (n=45), and their general characteristics are presented in Table 1. All the included studies (45/45, 100%) were categorized into different groups depending on the focus area of the mobile apps. The categories are oral health promotion, diagnostic, orthodontic, behavior management, trauma, and other.

**Table 1:** General characteristics of the included studies (n=45).

Characteristic			Studies, n (%)
**Year of publication**			
	2010-2019	22 (49)	
	2020-2021^a^	23 (51)	
**Category**			
	Oral health promotion	31 (69)	
	Diagnostic	1 (2)	
	Orthodontic	5 (11)	
	Behavior management	5 (11)	
	Trauma	2 (4)	
	Others	1 (2)	
**Study type**			
	Randomized controlled trial	23 (51)	
	Nonrandomized controlled trial and quasi-experimental study	5 (11)	
	Cross-sectional study	6 (13)	
	Longitudinal study	6 (13)	
	Case report	1 (2)	
	Systematic review	4 (9)	

^a^June 2021.

### Characteristics of the Included Studies

**Figure 2:**
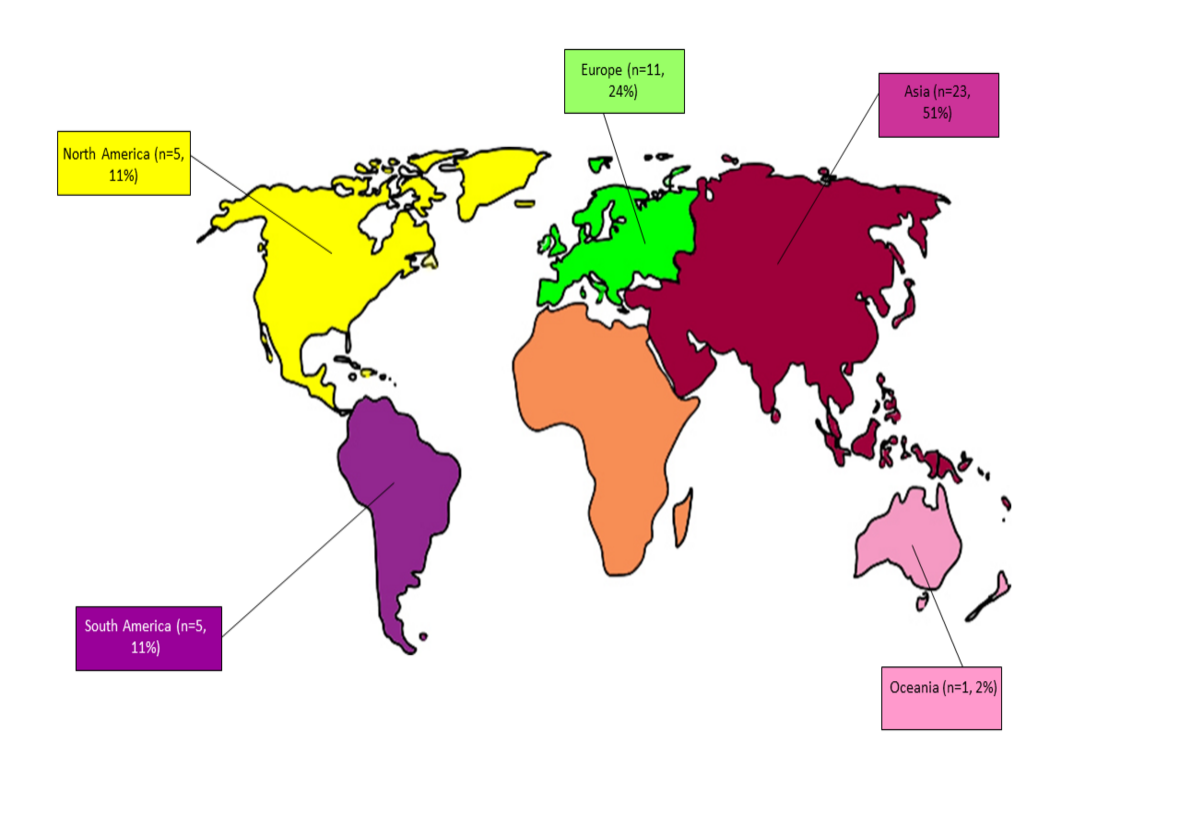
Geographical areas of the included studies (n=45).

Most studies (31/45, 69%) were categorized under oral health promotion. All studies (45/45, 100%) were published between 2009 and 2021. One-third (14/45, 31%) of the studies focused on adolescents aged 10 to 19 years, and 24% (11/45) of the studies focused on children aged <10 years. More than half (23/45, 51%) of the included studies were randomized controlled trials (RCTs). More than half (23/45, 51%) of the included studies were from Asia (Figure 2).

Table 2 shows the main findings of the included studies. Most of the included studies (21/45, 47%) focused on improving oral hygiene through effective toothbrushing. A total of 20% (9/45) of the studies focused on knowledge, attitudes, and practice [26-34]. In total, 11% (5/45) of the studies were based on treatment outcome [35-39], and 9% (4/45) of the studies reported on the use of mobile apps in the management of dental anxiety [40-43]. Only 2% (1/45) of the studies were based on disease diagnosis [44], and 2% (1/45) of the studies were based on remote dental care services [45]. Among the 45 studies, 4 (9%) systematic reviews were also included [46-49].

Approximately 39% (9/23) of the included RCT studies reported a significant reduction in dental plaque [26,46,53-57,59,63]. Similarly, 26% (6/23) of the studies reported significant improvement in gingival health measured via bleeding on probing [51,53-55,57,59], and only 4% (1/23) of the studies reported a reduction in enamel caries [59]. Regarding dental anxiety management, 75% (3/4) of RCT studies reported a significant decrease in mean heart rate and lower image scale scores compared with the controls [41-43].

**Table 2:** Main findings of the included studies (n=45).

Study, year	Country of origin	Study design	Sample size	Age	Purpose	Key findings
Alkilzy et al [50], 2019	Germany	Randomized controlled trial	60 recruited; 49 completed	Children aged 5-6 y	Investigate the efficacy of a manual toothbrush with a gravity sensor and mobile app for improving manual toothbrushing	At the 6- and 12-week follow-ups, the test group showed statistically significantly better oral health indexes than the controls. After the 6-week follow-up, the Quigley-Hein Plaque Index was 0.8 (SD 0.5) for the test group and 1.88 (SD 0.9) for the control group (*P*<.001), and the Papillary Bleeding Index was 0.08 (SD 0.1) for the test group and 0.26 (SD 0.2) for the control group (*P*<.001). After the 12-week follow-up, the Quigley-Hein Plaque Index was 0.44 (SD 0.5) for the test group and 1.49 (SD 0.7) for the control group (*P*<.001), and the Papillary Bleeding Index was 0.05 (SD 0.18) for the test group and 0.21 (SD 0.1) for the control group (*P*<.001).
Chang et al [51], 2021	Taiwan	Randomized controlled trial	150 recruited; 88 completed	26-77 y	Investigate the effectiveness of a mobile app (OSCA^a^) in improving OHS^b^ and OHBs^c^	After the 4- to 8-week follow-ups, no significant difference in OHS improvement measured using the O’Leary PCR^d^ was found between the control and intervention groups (mean OHS improvement was 17.0, SD 18.84 in the control group and 26.17, SD 21.76 in the intervention group; *P*=.06). OHBs (frequency of toothbrushing, duration of toothbrushing, interdental cleaning, and tongue cleaning) in both the control and intervention groups improved significantly (*P*=.007 and *P*<.001, respectively).
Alkadhi et al [52], 2017	Saudi Arabia	Randomized controlled trial	44	≥12 y	Investigate the impact of using mobile app active reminders to improve oral hygiene compared with verbal oral hygiene instructions	Both PI^e^ and GI^f^ significantly decreased after 4 weeks of using active reminders of oral hygiene instructions on a mobile app compared with verbal oral hygiene instructions (*P*=.04 and *P=*.02, respectively). For mobile app users, mean PI was 0.8007 (SD 0.4062) at baseline and 0.6677 (SD 0.3146) after 4 weeks, and mean GI was 0.3450 (SD 0.2955) at baseline and 0.2273 (SD 0.2256) after 4 weeks. For verbal oral hygiene instruction recipients, mean PI was 0.8959 (SD 0.4824) at baseline and 0.9891 (SD 0.5244) after 4 weeks, and mean GI was 0.4927 (SD 0.3005) at baseline and 0.5941 (SD 0.5679) after 4 weeks.
Farhadifard et al [53], 2020	Iran	Randomized controlled trial	120	15-25 y	Evaluate the efficacy of a smartphone app (Brush DJ) for oral hygiene compliance of patients with fixed orthodontic appliances	Significant improvements in PI and GI were observed in the group using the smartphone app (Brush DJ) compared with conventional oral hygiene instruction recipients after 4 weeks (T1), 8 weeks (T2), and 12 weeks (T3; *P*<.001). For mobile app users, mean PI was 75.21 (SD 13.36) at baseline, 73.39 (SD 12.50) at T1, 69.18 (SD 11.84) at T2, and 67.84 (SD 12.33) at T3, whereas the mean GI was 1.29 (SD 0.49) at baseline, 1.20 (SD 0.04) at T1, 1.04 (SD 0.04) at T2, and 1.00 (SD 0.05) at T3. For conventional oral hygiene instruction recipients, mean PI was 76.59 (SD 12.76) at baseline, 76.89 (SD 11.11) at T1, 78.90 (SD 8.89) at T2, and 80.82 (SD 10.05) at T3, whereas the mean GI was 1.49 (SD 0.59) at baseline, 1.35 (SD 0.04) at T1, 1.41 (SD 0.04) at T2, and 1.37 (SD 0.05) at T3.
Deleuse et al [54], 2020	Belgium	Randomized controlled trial	44 recruited; 38 completed	12-18 y	Compare the use of an oscillating electrical toothbrush with an internet-based oscillating electrical toothbrush connected to a brushing aid app in adolescent patients treated with fixed multibracket orthodontic appliances	PI and GI decreased significantly in both the control and intervention groups. WSL^g^ score was stable for both groups. PI was significantly lower in the app group than in the control group (*P*=.01) after 12 weeks. GI decreased significantly in each group (control group: *P*=.003; test group: *P*=.001), and no difference was observed between the 2 groups. WSL scores remained stable in each group (control group: *P*=.07; test group: *P*=.73), and no difference was observed between the 2 groups (*P*=.28)
Desai et al [55], 2021	India	Randomized controlled trial	247	4-6 y	Test the impact of a mobile app (Brush Up) on OHB in children	After the 1-month follow-up, plaque scores measured using the visible biofilm index was lower in the mobile app group (mean rank scores were 112.5 and 70.9 at baseline and follow-up, respectively) than those of the video demonstration group (mean rank scores were 114.8 and 112.5 at baseline and follow-up, respectively) and the manual demonstration group (mean rank scores were 134.8 and 176.8 at baseline and follow-up, respectively; *P*<.001). There was also significant change in the frequency and duration of toothbrushing, cleaning of the lingual surfaces of the teeth, and tongue cleaning in all groups (*P*<.001).
Kay and Shou [56], 2019	United Kingdom	Randomized controlled trial	108 recruited; 103 completed	18-69 y	Investigate the effectiveness and acceptability of a smartphone app used in conjunction with a movement sensor toothbrushing attachment (device) for reducing plaque levels	After the 4-week follow-up, mean full mouth plaque scores declined from 40.1 to 11.7 in the test group compared with a reduction from 29.1 to 20.5 in the control group (*P*<.001).
Marchetti et al [26], 2018	Brazil	Randomized controlled trial	291 recruited; 263 completed	14-19 y	Study the effectiveness of an app associated with common education methods in adolescents’ oral health	After the 1-month follow-up, a significant difference in mean KS^h^ between the adolescents who used the app (mean 4.77, SD 0.52) and those who did not (mean 4.35, SD 0.66; *P*<.001) was observed, and OHI-S^i^ decreased significantly among app users (mean OHI-S scores were 1.31, SD 0.37 at phase I and 0.24, SD 0.18 at phase IV, with *P*<.001, for the oral guidance plus app arm and 1.21, SD 0.39 at phase I and 0.23, SD 0.22 at phase IV, with *P*<.001, for the video guidance plus app arm). GBI^j^ decreased significantly among app users (mean GBI scores were 11.57, SD 5.09 at phase I and 2.03, SD 1.56 at phase IV, with *P*<.001, for the oral guidance plus app arm and 9.76, SD 4.07 at phase I and 1.87, SD 2.23 at phase IV, with *P*<.001, for the video guidance plus app arm).
Shida et al [57], 2020	Japan	Randomized controlled trial	118 recruited; 112 completed	≥18 y	Compare the effectiveness between brushing teeth with the help of a mobile app and usual brushing instructions	The mean 6-point PCR score at week 4 was 45.05% in the intervention group and 49.65% in the control group. The change of PCR score from baseline was −20.46% in the intervention group and −15.77% in the control group, indicating no statistically significant difference (95% CI −0.70 to 10.07; *P*=.09).
Zotti et al [58], 2016	Italy	Randomized controlled trial	80	Control group: mean age 13.6 y; study group: mean age 14.1 y	Study the effectiveness of the use of a mobile app via chat room participation (WhatsApp) to improve oral hygiene in adolescents wearing fixed appliances	After 6, 9, and 12 months, study group patients had significantly lower scores for both PI and GI and a lower incidence of new WSs^k^ compared with the control group. Mean PI scores at 12 months were 1.06 (SD 0.47) for the study group and 1.79 (SD 0.54) for the control group (*P*<.001). Mean GI scores at 12 months were 0.67 (SD 0.36) for the study group and 1.40 (SD 0.57) for the control group (*P*<.001). The number of patients with WSs was 7 for the study group and 16 for the control group (*P*<.05).
Scheerman et al [59], 2020	The Netherlands	Randomized controlled trial	132 recruited; 124 completed	12-16 y	Study the effectiveness of the use of a mobile app (WhiteTeeth) to improve oral hygiene in adolescent patients with fixed orthodontic appliances	At the 6-week follow-up, the intervention led to a significant decrease in gingival bleeding (B=−3.74, 95% CI −6.84 to −0.65; *P*=.02) and an increase in the use of fluoride mouth rinse (B=1.93, 95% CI 0.36-3.50; *P*=.02). At the 12-week follow-up, dental plaque accumulation (B=−11.32, 95% CI −20.57 to −2.07; *P*=.02) and the number of sites covered with plaque (B=−6.77, 95% CI −11.67 to −1.87; *P*=.007) had decreased significantly more in the intervention group than in the control group.
Patil et al [46], 2021	Saudi Arabia	Systematic review	N/A^l^	N/A	Review the effectiveness of the use of mobile apps to improve oral hygiene in patients with orthodontic appliances	Mobile apps have a significant short-term effect for improving oral hygiene when measuring using PI and GI scores. The intervention groups (62%) had a lower level of plaque at a 12-week interval as compared with the control group (72%).
Underwood et al [60], 2015	United Kingdom	Cross-sectional survey	189	All age groups	Evaluate the user experience of using a mobile app (Brush DJ) to provide a basis for future research	A total of 70% of respondents reported that their teeth felt cleaner since using the app. A total of 88% of respondents reported that the app motivated them to brush their teeth for longer. A total of 92.3% of respondents would recommend the app to their friends and family.
Toniazzo et al [47], 2019	Brazil	Systematic review and meta-analysis	N/A	N/A	Assess the effectiveness of the use of mobile apps and SMS text messages and compare them with conventional oral hygiene instructions to improve oral hygiene	The use of mobile apps and SMS text messages significantly improved oral health compared with conventional oral hygiene instructions. The pooled SMD^m^ for the PI was −9.43 (95% CI −14.36 to −4.495; *I*^2^=99%; *P*<.001), and that of gingival bleeding was −8.54 (95% CI −13.16 to −3.91; *I*^2^=99%; *P*<.001).
Rasmus et al [61], 2021	Finland	Longitudinal study (follow-up)	36	Children aged 4-12 y and their parents	Investigate the acceptability of a mobile app (Denny the Tooth and Denny the Timer) and evaluate OHB change	After the 5-week follow-up, most of the children considered the Denny the Tooth app clear (n=34), amusing (n=31), and useful (n=29). Denny the Timer was useful, and the odds for toothbrushing frequency significantly increased (OR^n^ 8.9, 95% CI 1.29-60.60; *P*=.03).
Scheerman et al [62], 2020	Iran	Randomized controlled trial	791 recruited; 718 completed	12-17 y	Study the effectiveness of a mobile app (Telegram) to promote and improve OHB	Increases in adolescent toothbrushing at the 1- and 6-month follow-ups in both intervention groups (adolescents only [A] and adolescents and their mothers [M+A]) compared with the control group were observed (1-month follow-up: B=3.74, SE 0.28, and *P*<.001 for the M+A group and B=2.64, SE 0.29, and *P*<.001 for the A group; 6-month follow-up: B=3.90, SE 0.27, and *P*<.001 for the M+A group and B=2.78, SE 0.29, and *P*<.001 for the A group). Adolescents in both intervention groups showed a significantly greater improvement in their VPI^o^ scores than adolescents in the control group at the 1- and 6-month follow-ups (*P*<.01; 1-month follow-up: B=−0.60, SE 0.05, and *P*<.001 for the M+A group and B=−0.29, SE 0.07, and *P*<.001 for the A group; 6-month follow-up: B=−0.64, SE 0.08, and *P*<.001 for the M+A group and B=−0.33, SE 0.06, and *P*<.001 for the A group).
Humm et al [63], 2020	Switzerland	Randomized controlled trial	20	≥18 y	Determine whether a smartphone app used with an electric toothbrush improves plaque removal compared with the use of an electric toothbrush without the app; in addition, the compliance and consideration of user-friendly were evaluated	No relevant difference in plaque score was found between the test and control groups (*P*=.39). However, PI improved by 8.5% (*P*=.10) in the intervention group compared with 4.7% (*P*=.56) in the control group.
Alqarni et al [27], 2018	Saudi Arabia	Longitudinal study	120	Parents of infants to adolescents aged 15 y	Develop a mobile app (Your child’s smile) and evaluate its efficacy in improving the dental health knowledge of parents	After the 15-day follow-up, most responders showed highly significant (*P*<.01) or significant (*P*<.05) improvement in their knowledge on tooth development (8.33%-40%), importance of deciduous teeth (25%-33%), importance of regular dental checkups (20%-34%), pit and fissure sealants (24%-32%), and consequences of early loss of deciduous teeth (19%-37%). A total of 75% of parents favored the use of mobile apps as an effective child dental health knowledge tool.
Zahid et al [64], 2020	Saudi Arabia	Nonrandomized quasi-experimental study	271 recruited; 234 completed	Mean age 16.6 (SD 0.96) y	Compare the impact of a mobile app (Brush DJ) and an educational lecture on knowledge and behavior regarding oral health	After the 3-month follow-up, both the mobile app and educational lecture groups showed significant improvements on knowledge and behavior regarding oral health except for the frequency and duration of toothbrushing in the app group.
Krishnan et al [65], 2021	India	Nonrandomized controlled trial	60	13-17 y	Evaluate the effectiveness of visual cards and a mobile-based app (Brush Up) on oral health education among adolescents with ASD^p^	At 6 and 12 weeks after the intervention, no statistically significant difference in PI (1.023 and 0.812; *P*=.91) and GI scores (0.264 and 0.283; *P*=.93) between visual pedagogy (group A) and the Brush Up mobile app (group B) was observed. For group A, mean plaque scores were 2.02 (SD 0.06) at baseline, 1.00 (SD 0.00) at 6 weeks, and 0.45 (SD 0.13) at the 12-week follow-up. Mean GI scores were 1.05 (SD 0.13) at baseline, 0.61 (SD 0.14) at 6 weeks, and 0.28 (0.10) at the 12-week follow-up. For group B, mean plaque scores were 2.01 (SD 0.06) at baseline, 1.00 (SD 0.00) at 6 weeks, and 0.46 (SD 0.11) at the 12-week follow-up. Mean GI scores were 1.03 (SD 0.17) at baseline, 0.58 (SD 0.16) at 6 weeks, and 0.24 (SD 0.11) at the 12-week follow-up.
Setijanto et al [28], 2021	Indonesia	Longitudinal study	47	17-45 y	Evaluate the effectiveness of a mobile app to increase knowledge about oral health among pregnant women	There was a significant (*P*<.001) improvement in the knowledge about oral health at posttest measurement (87%) compared with at pretest measurement (56%).
Fernández et al [48], 2021	Chile	Systematic review and meta-analysis	N/A	N/A	Determine the effect of teledentistry on oral health promotion and prevention as compared with other conventional strategies	Teledentistry was found effective—mostly mHealth^q^ (messages and apps)—when compared with conventional strategies. SMD for PI was −1.18 (95% CI −1.54 to −0.82; *I*^2^=92%; low certainty). SMD for GI was −2.17 (95% CI −3.15 to −1.19; *I*^2^=97%; moderate certainty). Risk ratio for WSLs was 0.48 (95% CI 0.35-0.66; *I*^2^=0%; moderate certainty).
Bohn et al [29], 2018	United States	Cross-sectional study	25	22-89 y	To assess preferences and perceptions regarding the use of apps in dental care	Participants believed that apps should be used in conjunction with a dentist’s explanation about a procedure. Participants felt that the apps would be more beneficial if they could be customized to individual dental needs. Participants favored esthetic images of teeth that did not show structural anatomy. Participants preferred the internet-based apps.
Rahaei et al [66], 2022	Iran	Randomized controlled trial	158	10-12 y	Compare the effectiveness of an educational mobile app (My Tooth) with conventional oral health education among elementary school students	Before the intervention, the mean scores of behavior were 13.69 (SD 3.89; intervention group) and 13.93 (SD 3.02; control group). After the intervention, mean scores of behavior increased significantly in the intervention group (16.02, SD 3.48; *P*<.001).
Nayak et al [30], 2018	India	Randomized controlled trial	159 recruited; 150 completed	18-24 y	Evaluate the feasibility and effectiveness of a mobile app (WhatsApp) to improve knowledge about oral cancer among college students	After the 1-month intervention, a statistically significant increase in KS was observed in both groups, with highly significant improvement in the intervention group (mean KS was 28.72, SD 5.9 at baseline and 49.56, SD 5.4 after the intervention; *P*<.001).
Zolfaghari et al [67], 2021	Iran	Randomized controlled trial	58	Children aged 1-6 y and their parents	Design a gamified mobile app and evaluate its effectiveness on educating mothers about their children’s oral health	The mean KS of mothers in the pretest was 10.5 (SD 2.1) in the simple app group and 11.3 (SD 11.9) in the gamified app group, which changed to 13.1 (SD 1.6) and 14.3 (SD 2.0), respectively, in the posttest (*P*<.001). The mean practice score of mothers in the pretest was 4.4 (SD 2.4) in the simple app group and 4.8 (SD 3.2) in the gamified app group, which changed to 8.5 (SD 1.7) and 8.0 (SD 2.2), respectively, in the posttest (*P*<.001). The mean dental PI of children in the pretest was 0.8 (SD 0.4) in the simple app group and 1.0 (SD 0.3) in the gamified app group, which changed to 0.5 (SD 0.3) and 0.5 (SD 0.3), respectively, in the posttest. Children had better plaque control in the gamified app group (*P*<.001).
Panchal et al [68], 2017	India	Longitudinal study	150 recruited; 132 completed	Children aged 2-6 y and their parents	Study the efficiency of a mobile app (Cariometer) in monitoring diet and oral hygiene habits	There was a significant improvement in the dietary pattern followed by the patients at day 7 as compared with day 1 (mean dietary score was 1.51, SD 0.24 at day 1 and 0.07, SD 0.14 at day 7; *P*<.001). Approximately 90% of children brushed twice a day at day 7 after the use of the Cariometer app. There was an increase in the frequency of rinsing after meals at day 7 as compared with day 1 after the use of the Cariometer app.
Lotto et al [31], 2020	Brazil	Randomized controlled trial	104	Children aged 36-60 months and their parents	Assess the efficiency of educational messages via mobile app (WhatsApp) to control early childhood caries	Proportion of participants with the increment of maximum ICDAS^r^ did not increase significantly in the intervention group (15.4%-23.1%; *P*=.13), differently from that observed in the control group (21.2%-36.5%; *P*=.008) between the 3- and 6-month follow-ups. eHEALS^s^ scores increased significantly in the intervention group (+10.32%; *P*=.001) in contrast to a nonsignificant decrease observed in the control group (−2.65%; *P*=.38).
Wang et al [32], 2020	Taiwan	Nonrandomized controlled trial	120 recruited; 100 completed	48-66 y	Evaluate an educational mobile app regarding changes in the care needs and quality of life of patients with oral cancer	The overall improvement in quality of life was higher in the experimental group than in the control group (−7.24 vs −4.36; *P*=.22). The physiological care needs decreased in the experimental group compared with the control group (experimental group: 26.33 and control group: 21.33 before the intervention; experimental group: 20.67 and control group: 20.25 after the intervention; *P*<.02).
Lozoya et al [69], 2019	United States	Nonrandomized quasi-experimental study	33 recruited; 26 completed	Children (mean age 3.48, SD 0.93 y) and their parents	Evaluate the effect of a smartphone app on the OHBs of the parents of preschoolers	Parents’ behavioral intentions or OHBs with their children did not significantly change from before to after the intervention (*P*>.05). SNs^t^ and PBC^u^ predicted behavioral intentions before the intervention and behavior change after the intervention. Thematic analysis revealed that parents’ belief in the importance of establishing oral health habits and brushing reminders and videos delivered via a mobile app supported efforts to form oral health habits.
Jacobson et al [70], 2019	United States	Quasi-experimental study	34	Children aged 5-6 y and their parents	Evaluate a mobile app (Brush Up) to improve toothbrushing behaviors among children	After 7 days, toothbrushing duration increased significantly (*P*<.001). Mean time (in seconds) consumed toothbrushing was 46.2 (SD 31.3) at baseline and 69.4 (SD 30.4) after 7 days (*P*<.001). After 2 weeks (n=15), mean time (in seconds) consumed toothbrushing was 39.9 (SD 21.0) at baseline and 108.9 (SD 47.6) after 14 days (*P*<.001).
Tobias and Spanier [44], 2020	Israel	Longitudinal study	44	≥18 y	Classification of gum health based on the MGI^v^ score using dental selfies via a mobile app (iGAM^w^).	The mobile app produced accurate classification of gum health based on the MGI. Area under the curve ranged between 1.0 and 0.84.
Al-Moghrabi et al [35], 2020	United Kingdom	Randomized controlled trial	84 recruited; 64 completed	12-21 y	Assess the effectiveness of a mobile app (My Retainers) on objectively assessed TPR^w^ wear time, stability, periodontal outcomes, patient experiences, and knowledge related to retainers	Use of the mobile app resulted in slightly higher median wear time (0.91 h/d, 95% CI −4.01 to 2.19; *P*=.56). No significant differences were found in terms of stability (β=.002, 95% CI −.03 to .04; *P*=.92), plaque levels (β=−.02, 95% CI −.07 to .03; *P*=.44), bleeding on probing (β=−.01, 95% CI −.05 to .03; *P*=.61), and probing depth (β=−.01, 95% CI −.09 to .07; *P*=.79). Similar levels of patient experiences (*P*=.94) and knowledge related to retainers (*P*=.26) were found.
Moylan et al [36], 2019	United States	Cross-sectional study	12	10-17 y	Study the reliability and accuracy of mobile app monitoring of tooth movement in patients with orthodontic appliances	The intercanine and intermolar measurement differences between intraoral video scans using the monitoring software’s smartphone app (Dental Monitoring) and plaster models were on average 0.17 mm (90% CI 0.00-0.34) and −0.02 mm (90% CI −0.26 to 0.29), respectively.
Li et al [37], 2016	China	Randomized controlled trial	343 recruited; 224 completed	Mean age 17.6 (SD 5.7) y	Evaluate the effectiveness of a messaging mobile app (WeChat) in improving patients’ compliance and decreasing the length of orthodontic treatment	Duration of orthodontic treatment in the WeChat group was shorter than that in the compared group (median 80.5, range 66-93 weeks vs median 84.5, range 75-103 weeks; *P*=.007). There was less failed attendance (3.1% vs 10.9%; *P*<.001), late attendance (20.1% vs 29.9%; *P*<.001), and bracket bond failure (11.8% vs 16.1%; *P*<.001) in the WeChat group than in the control group. There was no difference in orthodontic PI or MGI between the 2 groups before and after treatment.
Hannequin et al [38], 2020	France	Case report	N/A	21 y	Use dental monitoring software to manage aligner-mediated tooth movement on a woman aged 21 years treated with corticotomy-accelerated presurgical decompensation with Invisalign clear aligners	The software allowed for fewer chairside appointments and remote monitoring and allowed for early detection and correction of an error on the aligner. This case was managed in 6 months instead of 10-15 months for the presurgical decompensation phase and 3-4 months after surgery in conventional orthodontic treatments.
Henzell et al [39], 2013	New Zealand	Cross-sectional study	130	≥10 y	Investigate the use of internet-based social media sites by patients with orthodontic appliances and whether a web-based application or mobile app would be considered helpful in improving cooperation in orthodontic treatment	Internet-based social media sites were used by 80.8% of patients, with Facebook being the most popular. Approximately 13.3% of the sample had posted comments about braces on these social media sites, and only 6.7% had considered obtaining information about orthodontic treatment from internet-based social media sites, with most (81%) preferring to seek this information directly from their orthodontist. Nearly two-thirds of those who had difficulty remembering to wear their orthodontic appliances reported that a reminder app on their phone would be beneficial.
Al-Musawi et al [33], 2017	Kuwait	Longitudinal study	87	Not reported	Evaluate the effectiveness of a mobile app (Dental Trauma App) in delivering information to schoolteachers about the optimal emergency management of traumatic dental injuries and compare it with the traditional lecture-based method	Participants using the app only had a significantly higher mean score (12.72, SE 0.47) than participants receiving the lecture only (mean 11.20, SE 0.44; *P*=.02) and participants in the lecture and app group (mean 9.87, SE 0.50; *P*<.001).
Iskander et al [34], 2016	United States	Cross-sectional study	89	Not reported	Compare effectiveness and user preference of a mobile app and conventional poster for first aid to dental trauma	Individuals using the mobile app were more likely to select “put the tooth back in place” (71.1%) compared with those using the poster, who chose “put the tooth in milk” (56.8%; *P*=.004). Less educated individuals were willing to pay more for the app (*P*=.02) and were more likely to report being interested in receiving dental information through mobile technology in the future (*P*=.006). Most of the respondents preferred the mobile app.
Abbasi et al [40], 2021	Pakistan	Randomized controlled trial	160	Children aged 4-10 y	Evaluate the effectiveness of a mobile app (Little Lovely Dentist), a dental song, and TSD^x^ techniques in pediatric patients	A statistically significant difference in mean heart rate of participants was observed in the Little Lovely Dentist, dental song, and control groups, whereas no difference was observed in the TSD group. For the mobile “Little Lovely Dentist” app group, mean heart rate before and after the intervention was 107.9 (SD 8.2) and 104.9 (SD 6.8), respectively (*P*=.002). For the YouTube “dental video song” group, mean heart rate before and after the intervention was 106.6 (SD 6.1) and 104.0 (SD 7.6), respectively (*P*=.001). For the TSD group, mean heart rate before and after the intervention was 101.4 (SD 15.6) and 108.2 (SD 7.5), respectively (*P*=.68). For the control group, mean heart rate before and after the intervention was 102.8 (SD 5.3) and 107.5 (SD 5.9), respectively (*P*=.68). FIS^y^ scores decreased significantly in participants in the Little Lovely Dentist and dental song groups, whereas the scores increased in the TSD and control groups. For the mobile “Little Lovely Dentist” app group, mean FIS scores before and after the intervention were 2.80 (SD 1.06) and 2.52 (SD 0.87), respectively (*P*=.03). For the YouTube “dental video song” group, mean FIS scores before and after the intervention were 1.80 (SD 0.96) and 2.47 (SD 0.84), respectively (*P*=.04). For the TSD group, mean FIS scores before and after the intervention were 2.60 (SD 0.74) and 3.30 (SD 0.96), respectively (*P*=.001). For the control group, mean FIS scores before and after the intervention were 2.91 (SD 0.92) and 3.52 (SD 1.24), respectively (*P*=.01).
Elicherla et al [41], 2019	India	Randomized controlled trial	50	7-11 y	Evaluate the effectiveness of a mobile app (Little Lovely Dentist) compared with the TSD technique in managing anxiety and fear in children during their first dental visit	A statistically significant difference in mean heart rate of the participants was observed in the Little Lovely Dentist group, whereas no difference was observed in the TSD group. For the mobile “Little Lovely Dentist” app group, mean heart rate before and after the intervention was 108.2 (SD 12.8) and 97.4 (SD 12.3), respectively (*P*<.001). For the TSD group, mean heart rate before and after the intervention was 95.9 (SD 10.0) and 97.2 (SD 12.3), respectively (*P*=.32). RMS pictorial scores decreased significantly in participants in both the Little Lovely Dentist and TSD groups. For the mobile “Little Lovely Dentist” app group, mean RMS pictorial scores before and after the intervention were 3.20 (SD 1.04) and 1.32 (SD 0.5), respectively (*P*<.001). For the TSD group, mean RMS pictorial scores before and after the intervention were 2.6 (SD 0.8) and 1.5 (SD 0.6), respectively (*P*<.001).
Kevadia et al [42], 2020	India	Randomized controlled trial	75	6-9 y	To evaluate the effectiveness of 3 different behavioral management techniques: a mobile app (My Little Dentist), Tell, Play, Do, and the film modeling technique	All the index scores were significantly lower among children who received the Tell, Play, Do (group II) intervention than in those who received the film modeling intervention (group I) and the mobile dental app (group III). Average heart rate was *P*=.03 for group II vs group I and *P*=.046 for group II vs group III. FIS was *P*=.03 for group II vs group I and *P*=.03 for group II vs group III. Venham pictorial index scores were *P*=.04 for group II vs group I and *P*=.045 for group II vs group III.
Zink et al [43], 2018	Brazil	Randomized controlled trial	40	9-15 y	Development and evaluation of a mobile app for patient-professional communication during dental visits of patients with ASD	The decayed, missing, and filled primary and permanent teeth index was similar for both groups (*P*=.60), being 1.5 (SD 3.0) for the app group and 0.7 (SD 1.3) for the Picture Exchange Communication System group. There were statistically significant differences in the number of attempts required for the pictures to acquire each skill proposed (room, ground, dentist, and 3-in-1 air and water syringe; *P*<.05) between the 2 groups, which were lower for the app group.
Cunningham et al [49], 2021	United Kingdom	Systematic review	N/A	N/A	To examine and identify studies that apply virtual reality or bespoke smartphone apps in dentistry to decrease patient anxiety and to study the effectiveness of these apps	In total, 3 studies using virtual reality in a dental setting demonstrated decreased pain and anxiety compared with no intervention. A fourth study used a bespoke dental app and imagery to prepare patients with ASD for dental treatment, finding statistically significant decreases in both the number of appointments and number of attempts required to carry out a procedure.
Lin et al [45], 2014	Taiwan	Cross-sectional study	26 dentists and 32 patients	Not reported	To improve dental care services using a mobile app (Dental Calendar) combined with cloud service	Results of assessments through interviews and questionnaires indicated a significant increase (*P*<.05) in both dentists’ and patients’ overall experiences. Total mean increment in after and before test for dentists was 5.875 (95% CI 1.968-9.782; *P*=.009). Total mean increment in after-before test for patients was 18.500 (95% CI 13.625-23.375; *P*<.001). Patients’ ability to reschedule appointments for sudden worse prostheses was 0.385 (95% CI 0.081-0.689; *P*=.02). Appointment reminding was 0.844 (95% CI 0.242-1.445; *P*=.007), appointment rescheduling for sudden worse prostheses was 0.781 (95% CI 0.204-1.359; *P*=.01), and dentist-patient relationship was 0.500 (95% CI 0.007-0.993; *P*=.047)

^a^OSCA: oral self-care mobile app.

^b^OHS: oral hygiene status.

^c^OHB: oral hygiene behavior.

^d^PCR: plaque control record.

^e^PI: Plaque Index.

^f^GI: Gingival Index.

^g^WSL: white spot lesion.

^h^KS: knowledge score.

^i^OHI-S: Simplified Oral Hygiene Index.

^j^GBI: Gingival Bleeding Index.

^k^WS: white spot.

^l^N/A: not applicable.

^m^SMD: standardized mean difference.

^n^OR: odds ratio.

^o^VPI: Visible Plaque Index.

^p^ASD: autism spectrum disorder.

^q^mHealth: mobile health.

^r^ICDAS: International Caries Detection and Assessment System.

^s^eHEALS: eHealth Literacy Scale.

^t^SN: social norm.

^u^PBC: perceived behavioral control.

^v^MGI: Modified Gingival Index.

^w^TPR: thermoplastic retainer.

^x^TSD: Tell, Show, Do.

^y^FIS: Facial Image Scale.

## Discussion

### Principal Findings

The aim of this study was to present the main findings in the literature on the use of mobile phone apps in oral health and evaluate the evidence available in the literature. This scoping review identified and reviewed 45 papers published between January 2000 and June 2021. Half (23/45, 51%) of the included studies were from Asian countries and focused on children and adolescents. In addition, most of the included studies (31/45, 69%) focused on oral health promotion using mobile phone apps, followed by behavior management. There was limited evidence of diagnostic and remote consultations using mobile phone apps.

A total of 47% (21/45) of the publications focused on improving oral hygiene through effective toothbrushing. Dental plaque is a biological component in the initiation of dental caries and periodontal diseases [71]. Therefore, it is important to remove dental plaque from tooth surfaces through daily toothbrushing with fluoridated toothpaste to prevent tooth decay and periodontal diseases [71]. This scoping review found that several of the RCT studies (9/23, 39%) reported a significant reduction in dental plaque among mobile phone app users compared with their controls. Hence, mobile phone apps can be used for oral health education and to teach patients good home care skills. A recent systematic review and meta-analysis showed good results in decreasing plaque, thereby improving gingival health and preventing the development of dental caries when both education and skills are incorporated [72].

In total, 11% (5/45) of the publications focused on behavior management, and in these publications, mobile apps were used to reduce the dental anxiety of patients. Dental anxiety usually develops in childhood, and its prevalence ranges from 5% to 20% among children [73,74]. Patients with dental anxiety can easily avoid or miss dental appointments and, therefore, might have poorer dental health [75]. New situations in dental clinics are unfamiliar to children. Mobile apps used beforehand are an effective tool to familiarize a child with dental appointments and instruments and help improve cooperation between a dentist and a child during dental treatments [43,49].

More than half (23/45, 51%) of the included studies were from Asia, especially from India. The population of Asia was 4.7 billion in 2022, and the 2 most populous countries in Asia are China (1.37 billion) and India (1.29 billion) [76]. Thus, the rate of mobile phone use is higher in Asia than in other continents. For the same reason, the production and use of mobile phone apps are also higher in Asia than in other continents. In addition, none of the included studies were published in Africa. This could be due to various challenges, such as infrastructure, scientific technologies, and resources in implementing mHealth interventions in Africa, as described by Kruse et al [77] in their systematic review. Regarding the European region, the General Data Protection Regulation law drafted and implemented by the European Union on May 25, 2018, may have affected the conduct of research that requires the processing of sensitive data (eg, oral health data) despite protecting patients’ privacy [78]. Cagnazzo [79] also argued that the overlap of the General Data Protection Regulation and the European Clinical Trials Regulation is creating a state of confusion among the scientific community. Furthermore, the author has argued about the bureaucratic issues related to follow-up clinical studies [79]. However, these aspects need further research.

This review also found that most of the included studies (25/45, 56%) focused on children (aged <10 years) and their families as well as on adolescents aged 10 to 19 years. The studies in this review did not include older adults as a focus group, which may be explained by the fact that the younger population has grown up with smartphones and is more familiar with using them. Using smartphones is part of adolescents’ daily routine, and smartphones tend to move around with them throughout the day. In addition, smartphones are highly valued by adolescents; they are easy to use and easily switched on [15]. Thus, approaching children and adolescents via smartphone is more convenient. In addition, the use of smartphones gives people the possibility to access information anywhere and anytime, and this increases patients’ autonomy and comfort [80].

Despite excluding studies focused on the development of mobile apps in dentistry, half (23/45, 51%) of the included studies were published between 2020 and 2021. This implies that the field is constantly evolving and growing. Furthermore, the COVID-19 pandemic started at the end of 2019 and changed dental care in many aspects. The use of teledentistry increased during the pandemic, and dentists with a clinical practice considered teledentistry to be the most effective way to reschedule patients’ appointment times and provide dental hygiene education and emergency advice [81,82]. In this scoping review, we found only 1 study [45] that focused on remote health care services. However, more studies on teledentistry and the use of artificial intelligence for diagnosis and remote consultations are necessary. In addition, topics related to the uses and importance of teledentistry and artificial intelligence must be added to the curriculum of undergraduate and postgraduate dental studies [83].

### Limitations

This study has some limitations. First, only studies written in English were included. Second, the search was carried out in only 2 databases (PubMed and Scopus), therefore leaving out literature from other databases and gray literature. However, a recent meta-research study suggests that searching at least 2 databases is sufficient to increase coverage and decrease the risk of missing eligible studies [84]. In addition, an information specialist was consulted for searching the databases. Another limitation would be not undergoing any peer-review for electronic search strategies.

The field of mobile apps is still new and under development. New research must be conducted along with the development of new patient-oriented apps.

### Conclusions

The use of mobile apps in oral health is increasing among patients, mainly children and adolescents. In the literature, there are many studies related to mobile apps that are focused on promoting oral health. Other areas such as diagnosis and remote consultations are neglected in the current studies. There are potential uses for improving oral hygiene, knowledge, and behavior via mobile apps, but more studies are required.

## Abbreviations

MeSH: Medical Subject Headings
mHealth: mobile health
PRISMA-ScR: Preferred Reporting Items for Systematic Reviews and Meta-Analyses extension for Scoping Reviews
RCT: randomized controlled trial
